# Cross-border data sharing through the lens of research ethics committee members in sub-Saharan Africa

**DOI:** 10.1371/journal.pone.0303828

**Published:** 2024-05-23

**Authors:** Nezerith Cengiz, Siti M. Kabanda, Keymanthri Moodley

**Affiliations:** Department of Medicine, Division for Medical Ethics and Law, Faculty of Medicine and Health Sciences, Stellenbosch University, Cape Town, South Africa; PLoS ONE, UNITED STATES

## Abstract

**Background:**

Several factors thwart successful data sharing—ambiguous or fragmented regulatory landscapes, conflicting institutional/researcher interests and varying levels of data science-related expertise are among these. Traditional ethics oversight mechanisms and practices may not be well placed to guarantee adequate research oversight given the unique challenges presented by digital technologies and artificial intelligence (AI). Data-intensive research has raised new, contextual ethics and legal challenges that are particularly relevant in an African research setting. Yet, no empirical research has been conducted to explore these challenges.

**Materials and methods:**

We explored REC members’ views and experiences on data sharing by conducting 20 semi-structured interviews online between June 2022 and February 2023. Using purposive sampling and snowballing, we recruited representatives across sub-Saharan Africa (SSA). We transcribed verbatim and thematically analysed the data with Atlas.ti V22.

**Results:**

Three dominant themes were identified: (i) experiences in reviewing data sharing protocols, (ii) perceptions of data transfer tools and (iii) ethical, legal and social challenges of data sharing. Several sub-themes emerged as: (i.a) frequency of and approaches used in reviewing data sharing protocols, (i.b) practical/technical challenges, (i.c) training, (ii.a) ideal structure of data transfer tools, (ii.b) key elements of data transfer tools, (ii.c) implementation level, (ii.d) key stakeholders in developing and reviewing a data transfer agreement (DTA), (iii.a) confidentiality and anonymity, (iii.b) consent, (iii.c) regulatory frameworks, and (iii.d) stigmatisation and discrimination.

**Conclusions:**

Our results indicated variability in REC members’ perceptions, suboptimal awareness of the existence of data protection laws and a unanimously expressed need for REC member training. To promote efficient data sharing within and across SSA, guidelines that incorporate ethical, legal and social elements need to be developed in consultation with relevant stakeholders and field experts, along with the training accreditation of REC members in the review of data-intensive protocols.

## Background

The volume of research generating primary data is increasing exponentially both globally [1] and in sub-Saharan Africa (SSA) [2]. The secondary use of data is also expanding as data sharing accelerates across the globe. Data is acknowledged as the new research currency and data sharing, which is the act of providing access by transferring data in a form that can be used by other individuals, is increasingly recognised for its several benefits. These have been well-documented to reflect research collaborations, resource preservation, knowledge advancement, enhanced data integrity, transparency, public accountability and improved patient-centred care through effective service delivery and informed policy decision-making [3–8]. However, ensuring fair and lawful data sharing remains challenging.

Research Ethics Committees (RECs) act as independent mediators between science and the public to ensure ethical and legal compliance. Their main purpose of protecting the dignity and rights of research participants is to foster social trust in research as well as assess its social value [9]. RECs play a pivotal role in the review and governance of all research including data-intensive research [10, 11]. However, traditional ethics oversight mechanisms and practices may not be well placed to guarantee adequate oversight of data-intensive research given the unique and novel challenges presented by digital technologies and artificial intelligence (AI) [12, 13].

The intrinsic exploratory and technical nature of data-intensive research poses challenges for RECs in adequately reviewing the data sharing aspects of research protocols such as the distinct methodology of studies that are based on data aggregation and mining which require specialised technical expertise [12]. Big data is important to develop algorithms for machine learning and AI. Given the evolving nature of AI, all potential challenges of data-intensive research may not be well-known at the time of protocol assessment by RECs, especially during early phases of the study such as the deployment phase of machine learning algorithms [12]. RECs are also challenged in assessing the scope of informed consent for data-intensive research as all possible future uses of big data such as AI development cannot be disclosed and may be unknown at the time of data generation. The content and type of consent, mechanisms of data sharing and appropriate community engagement efforts are also problematic [14]. The re-use of data poses unforeseeable risks and potential societal consequences, especially where broad consent is utilised or when data is collected from publicly accessible platforms allowing for additional data analysis without seeking data subject (re)consent and/or REC approval. In such cases, Data Access Committees (DACs) may be a viable option for ethical review given their technical expertise and knowledge of data governance equipping them to address key obstacles in AI and data-intensive research [15].

Furthermore, the evolving challenges consequently inflate the existing ethical gap as existing safety measures and research ethics guidelines may not apply to health-related data-intensive research [12]. Ethical governance, in conjunction with national data protection regulation, is necessary to foster responsible data sharing in data-intensive health research [16]. In particular, low-income and middle-income countries (LMICs) lack enforceable agreements, leaving researchers vulnerable to exploitation and failing to protect research participants from harm [17–19]. Consequently, agreements such as Data Transfer Agreements (DTA) and Material Transfer Agreements (MTA) are of paramount importance for RECs to scrutinise, ensuring compatibility with the research proposition in terms of informed consent or data transfer and sharing procedures [20].

In our previous study, we conducted the first empirical survey in SSA in which awareness and perspectives of REC members were explored specifically relating to the review of data-intense research protocols and data governance in SSA [11]. At the time, it was unclear how REC members navigate governance structures and processes and review such protocols. Our results indicated variability in REC members’ perceptions of the adequacy of their national laws and institutional policies, suboptimal awareness of the existence of data protection laws as well as a unanimous expressed need for REC member training.

We expanded on the previous study and delved deeper into these issues in this study—the first qualitative empirical study in SSA in which perspectives and experiences of REC members relating to reviewing data-intensive research protocols, data transfer tools as well as the ethical, legal and social challenges of data sharing in data-intensive studies are explored. This qualitative study is intended to provide context and depth to the issues identified in our quantitative study. Data-intensive research has raised new contextual ethics and legal challenges [21] making such investigation, particularly in the context of SSA, important to a wide range of stakeholders.

## Materials and methods

### Study design and sampling

We employed a qualitative exploratory study design using semi-structured interviews to explore REC members’ views on data sharing, overall experience with reviewing data-intense protocols that involved data sharing as well as their commonly faced challenges. We also explored their views on DTAs and DACs and the role that RECs play within these structures.

Data was collected from REC members from different countries in SSA–from the ground up to build concepts and theories. A grounded theory approach was used [22]. Hence, semi-structured interviews were conducted until theoretical saturation of data was reached. Flexibility, creativity and reflexivity were used in the interview process. The interviewer and interviewees were active in the construction of knowledge. There was prompting and probing during the interviews to better understand the challenges faced by REC members and how these challenges could be resolved. Flexibility was important as interviews were being conducted in different countries in SSA where data transfer laws, ethics guidelines and competence of REC members differed.

This study was conducted by a multi-disciplinary team of researchers. NC holds a Master’s degree in bioethics and has a background in Public health. SK holds a Master’s degree in Public Health and has a background in health sciences. KM is the study PI, a family physician with expertise in medical ethics, clinical research and research ethics. She has served on RECs for more than 10 years and holds a doctorate.

Our sample included representatives from 20 different SSA countries through a purposive selection of professional networks of the Stellenbosch University’s Division of Medical Ethics and Law across SSA. A snowballing technique was used for further recruitment. We also identified potential participants through a desktop search based on their profession and directly emailed REC members, inviting them to participate in their personal capacity. This study is linked to a previous descriptive cross-sectional online survey in which we invited interviewees to anonymously participate in an online survey through Research Electronic Data Capture (REDCap). At the end of the questionnaire, interviewees were asked to indicate their interest in participating in an in-depth interview. Of those who agreed and provided their email addresses (through anonymised branching logic which directed them to a Google Form), written informed consent forms were sent directly to them, signed and returned via email before the scheduled in-depth interviews. During the quantitative survey, some participants volunteered to participate in in-depth interviews for this study. Furthermore, some participants declined to participate due to time constraints.

### Data collection

From 02 June 2022 to 21 February 2023, we conducted 20 semi-structured, in-depth interviews with REC members via Microsoft Teams. All interviews were conducted virtually by NC, within a private space and without the presence of a third party. All interviews were conducted online, in English and lasted an average of 45 minutes. Verbal permission was obtained to record each interview via Microsoft Teams. Interview questions were formulated and adapted from Ferretti et al [12] and through discussion with members of the team. The interview guide was pre-tested with five REC members to assess readability, ensure clarity and refine the content before study commencement. Data was collected until saturation at a continental level was achieved given that most anglophone SSA countries had similar ethics challenges with respect to data sharing.

### Data analysis

Researchers transcribed verbatim the audio files and cleaned the data. The data was managed using Atlas.ti (version 22) software [23]. Within this software, the data was subjected to thematic analysis, employing an inductive approach. The data was coded and then grouped into subthemes which led to the generation of major themes. Adjustments to the final thematic map were made to improve logical cohesion through extensive deliberation among the team of involved members. The findings of this analysis are detailed in the results section. To achieve rigour in our findings and promote transparency of the coding process, intercoder reliability (ICR) was established through two researchers independently coding 20% of randomly selected transcripts followed by deliberations to reach a consensus on codes, generation of themes and any discrepancies. O’Connor and Joffe [24] recommend that 10–25% of data units should be coded to establish ICR.

### Ethical aspects

Research integrity was maintained throughout the study and participation remained entirely voluntary. We approached our sample in their individual capacity and they consented in their own personal capacity. The in-depth interviews were of minimal risk and ethics approval was granted by the Faculty of Medicine and Health Sciences Health REC (Reference No: N22/03/028) at Stellenbosch University, South Africa.

## Results

Our analysis identified three recurrent themes and several subthemes that are summarised below (Table 1).

**Table 1:** Overview of themes and sub-themes.

Theme	Sub-theme
1)Experiences in reviewing data sharing protocols	• Frequency of and approaches used in reviewing data sharing protocols
	• Practical/technical challenges faced in reviewing data sharing protocols
	• Need for training
2) Perceptions of data transfer tools	• Structure of data transfer tools
	• Key elements of data transfer tools
	• Level of implementation
	• Key stakeholders in developing and reviewing a DTA
3) Ethical, legal and social challenges of data sharing in research	• Confidentiality and anonymity
	• Consent
	• Regulatory frameworks
	• Stigmatisation and discrimination

### Demographic information

A total of 20 REC members from across SSA were interviewed and represented 20 of the 49 SSA countries (Table 2).

**Table 2:** Demographic characteristics of participants (n  =  20).

Country ID	Gender	Age group, years	REC type	Years of experience	Profession
Country 1	Male	40–49	National	1–6	Medical doctor/scientist
Country 2	Male	40–49	institutional	>13	Assistant Director/Ethicist
Country 3	Male	40–49	National	7–12	Professor/scientist
Country 4	Male	40–49	Institutional	1–6	Biologist
Country 5	Male	40–49	Institutional	1–6	Pharmacologist, researcher
Country 6	Female	50≥	Institutional	>13	clinical scientist, research ethicist
Country 7	Female	50≥	Institutional	7–12	Bioethicist
Country 8	Female	50≥	Institutional	Did not respond	Behavioural Scientist/Lecturer
Country 9	Female	30–39	National	7–12	Biomedical scientist
Country 10	Male	50≥	Institutional	1–6	Clinician/researcher
Country 11	Male	50≥	National	>13	Head of research office/scientist
Country 12	Male	50≥	Institutional	1–6	Research medical officer
Country 13	Male	50≥	Institutional	1–6	Lecturer/Ethicist
Country 14	Male	40–49	National	1–6	Social scientist/senior monitoring officer
Country 15	Male	40–49	Institutional	7–12	Scientist
Country 16	Male	40–49	National	1–6	Scientist
Country 17	Female	50≥	National	>13	Vet
Country 18	Female	40–49	Institutional	1–6	Scientist
Country 19	Female	40–49	Institutional	1–6	Research, Medical officer
Country 20	Male	30–39	Institutional	Did not respond	Medical doctor/Lecturer/scientist

Thirteen of the interviewees served in the institutional REC, while 7 served in the national REC. Eight of the 20 interviewees were female and the remaining 12 were male. The majority were between the ages of 40–49.

## Theme 1: Experiences in reviewing data sharing protocols

### Frequency of and approaches used in reviewing data sharing protocols

Although a few interviewees indicated that they had not reviewed data-intensive research protocols, most reported having previously reviewed such protocols.

“*…most of our studies are multi-country and involve collaborative research so*, *I think…about 50 per cent of protocols [that we reviewed] involve data sharing*.*”* [Country 5]

### Approaches used in reviewing data sharing protocols

Interviewees revealed that reviewing data sharing protocols involved assessing data sharing agreements, procedures and mechanisms put in place to regulate data transfer. Some respondents were able to discuss the overall reviewing of data sharing protocols while others were able to elaborate more on data sharing aspects in research protocols. This varied from country to country but mostly, data sharing aspects in research protocols were discussed. In data-intensive research countries, interviewees could discuss data sharing in greater depth. In countries where protocols are not yet related to big data, discussions revolved around data sharing aspects of regular research.

“*… for the protection of the participant*, *we look out for the Material Transfer Agreement (MTA)*, *the agreement between the two institutions*, *what has been stated in it and whether it coincides with the main proposal?*” [Country 9]

“*…during our review of protocols*, *we check what measures researchers are bringing into action to avoid any risk associated with data sharing*…*”* [Country 5]

### Practical/technical challenges faced in reviewing data sharing protocols

Interviewees reported several challenges encountered while reviewing data sharing protocols such as data access requests without ethical approval, data management issues, incompleteness of transfer agreements and the inadequacy of the contents of consent forms for data-intensive research. The concerns raised by the interviewees regarding the management of national data were primarily centred around the storage of data at a national level. In the context of conducting multi-country studies, REC members may encounter difficulties regarding the capacity off or data management in each country involved.

“*before asking for data*, *ensure you have institutional review body clearance to use the data ethically and adhere to ethical principles… one of the biggest issues is that researchers want to access the data before even developing the protocol…”* [Country 12]

“*…the multi-country studies are challenging around data storage and data analysis*, *especially around confidence in the country’s data management capacity*…*”* [Country 14]

### Need for training

Interviewees emphasised the importance of REC members receiving training to review data sharing protocols. This is because some of the interviewees felt that their committees lacked the necessary expertise to evaluate such protocols. Some of the interviewees suggested that a structure be put in place to ensure that REC members have minimal, obligatory training before they are appointed.

“*…I propose more training*, *more exposure and awareness of the ethical challenges and emerging issues of data sharing so that they can get more familiar with the issues and know how to address them when they are faced with such*.*”* [Country 9]

## Theme 2: Perceptions of data transfer tools

Interviewees displayed differences in their perceptions of the ideal transfer agreement in terms of structure, key elements, level of implementation and key stakeholders.

### DTA as an individual document or combined with MTA

We asked interviewees to expand on their idea of the ideal structure for data transfer documents. Participants favoured different ideas as reported below:

“*I think it’s better to have them [MTA and DTA] combined because if the material that we are referring to actually gave birth to the data that is being asked for*, *it would be reasonable to have them together as one generated the other*… .*”* [Country 13]

Another interviewee shared a similar view:

*[MTAs and DTAs] should be combined as material and its associated data are linked*. *There are times when people ask for purely data transfers and there are also times when it is purely material transfers that are done…”* [Country 1]

On the other hand, some interviewees reasoned that because these documents cover different matters, they should not be collated or merged.

“*I think it would be good for them to be separate because they’re dealing with different things*. *If they were combined*, *then we might lose out on something*.*”* [Country 7]

“*DTAs and MTAs deal with two separate issues and should remain as two separate documents… combining them would reduce its rigour*. *People may cross over one aspect and focus on another*.*”* [Country 15]

### Key elements of data transfer tools

Interviewees further explained what they thought were some of the key characteristics that a data transfer agreement needed to comprise. Several ideas were noted such as the purpose of the data transfer, access control, location and duration of storage and potential for collaboration.

“*…sharing the data for what purposes*, *the type of data being shared with people…And the last part is the publication*. *We need really to mention how publication should be organised in the case of data sharing*.*”* [Country 16]

“*…It should cover what the data will be used for…how long it will be stored and how it will be disposed of*?*”* [Country 7]

“*…What are the research benefits of that data transfer*? *[Researchers] just want to transfer data and then nothing happens*, *we need to see the impacts of transferring that data*…*”* [Country 12]

Other interviewees raised some appealing considerations for a data transfer agreement suxh as accountability for data anonymisation and protection as well as mandatory monitoring and evaluation of data transfers to ensure adherence to its initial intended purposes.

“*…signing a commitment form to ensure that data will be protected… anonymity will be maintained… and the agreed terms and conditions… There should be some measures to monitor*. *It is not just about transferring data or sharing data*.*”* [Country 9]

### Level of implementation

Interviewees were split between whether a national or an institutional level DTA would be most impactful. Interviewees in favour of a national DTA argued that having a single, standard DTA would resolve inequities and limitations that may arise from having several varying data sharing agreements, each conforming to different institutional ideals regarding data access, restrictions and regulatory frameworks etc. Interviewees added that a national DTA may reduce any reliance on the guidelines and frameworks of other countries.

“*… Having one standardised data sharing agreement and one standardised material transfer agreement can resolve many challenges*, *inequities and inappropriateness that might exist in having many different data sharing agreements*…*”* [Country 3]

On the other hand, a few interviewees expressed support for an institutional level DTA and justified that a national-level DTA would be limited. One interviewee mentioned:

“*…[DTAs] would be best at an institutional level or even between research groups*. *I would be quite sceptical of a data agreement on a national level because it’s almost impossible to plug all the gaps*… *Data sharing at a country level will need legal advice*, *clarity on what types of data will be shared*, *how it’ll be used*, *by whom and for what…”* [Country 19]

### Key stakeholders in developing and reviewing DTA

Interviewees added that RECs, researchers, research institutions, government officials and legal experts are key role players in collectively developing a national DTA. Interviewees also highly suggested community members from which samples or data are collected from as important informants who should have their voices heard in the development of a DTA.

“*I think researchers themselves should be involved…they practically share the data and are the ones that will be the most affected*…” [Country 6]

“*If there is a community leader or even a Community Advisory Board*, *I think they should be included as well or a traditional leader*, *depending on where the data would come from—which community or communities*?*”*. [Country 19]

Regarding the DTA review process, once it has been developed, some respondents stated that, because of the variety of skills among their members, RECs were ideal stakeholders to review DTAs; while others sided with legal entities or DACs. On the contrary, some interviewees expressed disagreement towards RECs reviewing DTAs as they felt that they are not skilled enough to evaluate such a document. These interviewees supported the idea of DACs reviewing DTAs as they serve to regulate who has access to data.

“*I would definitely support a DAC with proper legal input and with proper ethics input [as] the people that are generally on a REC will not have the skill to make sure that all these processes are in place*…*would be critical if we want to be research-intensive universities*, *we want to be more competitive*. *We must make data shareable*, *but we do that responsibly and then we need to put some effort and money and resources into such committees…can do this well…who can do it timeously*…*”* [Country 6]

However, a few interviewees expressed unfamiliarity with the concept and role of DACs.

“Well, I’m not really sure [what DACs are], but I think there should be some guidelines [that describe their role and purpose]…” [Country 4]

## Theme 3: Ethical, legal and social challenges of data sharing in research

Interviewees listed several benefits to data sharing in research which are comparable to those reported in the literature, including the potential for collaborative endeavours, resource conservation, knowledge advancement, robust study findings, improved decision-making and informed policy development. However, this theme focuses on the challenges that interviewees perceived to exist in data sharing that were relevant to their context.

### Regulatory frameworks

Despite some of the favourable outcomes relating to evidence-informed policymaking, other interviewees perceived legal challenges in this regard and indicated that their countries lacked regulatory frameworks for data sharing.

“*The absence of data sharing laws has placed us in a position where many people*, *especially outside of the country know a lot of things that they are not supposed to know*, *or rather unconfirmed information…”* [Country 14]

“*When you do not have regulatory frameworks regarding data sharing*, *like in my country’s case*, *then you are in trouble because you do not have anything of your own to use for guidance*. *You tend to rely on the other person who is asking for your permission to have access to your data… which is disadvantageous to those owning the data being shared…”* [Country 13]

The absence of regulatory frameworks for data sharing provided leeway for researchers from the Global North to engage in “helicopter research” as reported by some interviewees. This is further explained as Global South researchers being denied or restricted in their participation and contribution to research projects, ranging from its conception to publication of findings.

“*…some researchers outside the country come to collect data and while they wait for protocol approval*, *they include local people and local researchers*, *then when it comes to writing a manuscript*, *they publish without local names*… *this is really*, *completely unfair because if you come to my country*, *it is me that has to write about my country*. *It is me that must be a principal investigator because it is my country… People write a draft and send it to local researchers to review and even if you do not agree*, *they do not take it into account… this really annoys and unfortunately*, *we do not have laws for such issues…”* [Country 17]

This interviewee further highlighted the need for data sharing to be regulated as noted below:

“*…data sharing has to be really very*, *very controlled*.*”* [Country 17]

### Consent

Failure to achieve awareness of and prior consent regarding the sharing of data were identified as a significant issues by our interviewees. In certain cases, the research participants may lack knowledge of the fact that their data will be shared or utilised for alternative purposes. Several interviewees emphasised the necessity of informing research participants about this aspect during the process of obtaining consent.

“*The participant does not [always] know that his or her data is going to be shared or is going to be used for another purpose*. *You cannot trace the participants again to sign a consent [form]… and I do not know how it can be solved…”* [Country 3]

“*…it is expected that if you want to share data*, *appropriate permission should be taken… when this is not done*, *it will also lead to a legal issue*…” [Country 10]

Interviewees stressed the importance of using data only for its intended collected purpose unless further consent is obtained from the participant or data subject. One interviewee commented:

“*How do you ensure that the data is being used for exactly [the same purpose] it was collected for? Anything beyond the primary objective of collection requires informing participants*.*”* [Country 7]

“*…one experience we had [in our institution] involved [a student] who took some specimens from the hospital and actually transported them outside [the country]*. *He wanted to use that information for his PhD studies and consent was not taken*. *There were concerns [about whether] the patients would want to know about their material…”* [Country 7]

### Confidentiality and anonymity

Interviewees agreed that maintaining confidentiality is challenging when sharing data, with one interviewee emphasising the importance of proper research study designs:

“*If the original research team puts proper measures in place to protect participants through a proper study design where confidentiality is maintained from the beginning and data is shared only in de-identified format then there is no issue*…*”* [Country 19]

One interviewee raised the issue of anonymising data, particularly when working with sensitive information even if the data has been de-identified and further mentioned issues with genomics and digital technologies:

“*…We were requested to make all the transcripts available [from a study exploring the perceptions of people living with HIV] by the journal and we refused because it was not possible to completely anonymise the sensitive data*. *It is possible to identify them with some degree of accuracy and this would be harmful to the participants*…*I think a lot of us who got to learn computers later*, *and the Internet*, *you know*, *have an inherent distrust of*, *you know*, *who has access to data because we don’t fully understand how people can gain access to data…My fears are always for genetic information*. *Because*, *you know*, *we’ve seen internationally that if you have databases that can speak to one another that have lots of detailed information*, *then people can be linked to their genetic code*… *And I do fear that this data could be linked to the person that to the person*, *and that can have lots of implications for families*, *for communities”* [Country 6]*”*

### Stigmatisation and discrimination

Most interviewees cited stigmatisation and discrimination as potential social challenges to data sharing and described how sensitive data may not always be well protected.

“*…If the data is not well protected*, *then somebody can have access to that and can be able to tell that it is [person A] who provided this information and that person can be stigmatised or discriminated [against]*…” [Country 9]

“…*with genetic studies*, *there could be findings that have social implications when disseminated to people without appropriate precautions*. *It could lead to stigma*, *labelling and social problems causing scepticism in research participation*.” [Country 10]

## Discussion

Interviewees’ general descriptions of data sharing displayed a relatively fair understanding and awareness of the role of data governance and regulatory frameworks that help make data lawfully accessible to others. The demonstration of understanding and awareness of this concept is important as there is an increased demand placed on RECs to engage with protocols involving big data and AI-driven research that may need to be shared during collaborations [12]. Data sharing is an important part of open science as more institutions, journals, health research funders and governments emphasise the importance of open science and enforce open data policies intended to increase academic influences and promote scientific discovery and development for the greater benefit of the public [10, 25–27]. Openness in science contributes to greater social impact on the public and our interviewees recognised its several benefits which are consistent with previous research [17, 28–30]. These benefits encompassed the potential for collaborative endeavours, the capacity to conserve resources, the advancement of knowledge and the increased robustness of study findings which could all potentially inform policy and decision-making [17, 28–30]. Open science is associated with substantial benefits, especially in the context of LMICs in SSA, and requires continued support and safeguards to foster lawful data sharing [31].

Despite interviewees having some extent of experience in reviewing data-intensive research protocols involving some form of data sharing, many acknowledged a gap in their own knowledge and skills to effectively review such protocols given some of the complex and emerging research study designs. This finding concurs with that of Ferretti *et al* where REC members in Switzerland reported having limited experience in reviewing data-intensive research protocols which stretched their expertise [12]. Consequently, our study interviewees stressed the significance of developing training programmes and capacity development initiatives for REC members to improve the quality of scientific and ethical reviews. With the increasing popularity of open science practices and the generation of large datasets, it is imperative for RECs to fully embrace the big data era [32, 33]. Ferretti *et al* suggest that RECs strengthen their oversight mechanisms by training in data science and big data ethics as well as acquiring technical skills in data analysis methodologies and AI-enabled technologies [12, 34]. Although SSA is often faced with limited funding and resources, the investment in such REC training would be significantly beneficial for both RECs and researchers across the continent [35]. It would thus be reasonable to expect that both research institutions and research sponsors take the initiative to support and fund the training of their institutional RECs.

Despite the manifold benefits of open science and data sharing, a multitude of ethical challenges also persist. These challenges span from the acquisition of informed consent to the preservation of confidentiality and anonymity. Our interviewees articulated similar challenges that are comprehensively documented in the literature [12, 34, 36–40]. In sharing de-identified data, interviewees questioned the feasibility of guaranteeing confidentiality and anonymity to research participants in the context of emerging digital technologies and especially, in research involving sensitive data given the potential for reversing de-identification measures. While de-identification [41] refers to the removal of or concealment of explicit identifiers, anonymisation refers to the process of rendering data in a form where identification is implausible or impossible [40]. Similarly, pseudonymisation involves replacing data identifiers with artificial ones or pseudonyms. This means that the data can no longer be attributed to a specific data subject without the use of additional information [42].

However, a residual risk of re-identification exists where de-identified or pseudonymised data are used [40] as reported by interviewees who expressed concern about the viability of ensuring confidentiality and anonymity. If RECs fail to adequately evaluate the ethical acceptability of the research and if researchers fail to take appropriate measures to guard against the risk of re-identification, stigmatisation and discrimination of vulnerable populations may result. Interviewees added that protecting sensitive data in the era of AI and big data is even more challenging regardless of its de-identified, anonymised or pseudonymised state. Cheah *et al* reported similar results indicating that participants had reservations about sharing sensitive data due to fear of stigma and discrimination from their communities, even once de-identified [43]. This may be related to the increased risk of cyber-attacks and data breaches on healthcare databases where large volumes of personal health and identifiable information are kept [44]. Beyond stigma and discrimination, the impact of data breaches may further result in physical, financial, psychological and societal harm as well as dignity damages [44].

Sardanelli *et al*. conceded and cautioned that de-identified data do not eliminate all risks of re-identification and that reducing this risk to zero may lessen the data utility for subsequent analysis or verification [45]. It may also impact benefit sharing negatively if participants will not be receiving their study results due to anonymisation procedures [45, 46]. This consequence must be clearly explained to research participants to manage expectations and prevent therapeutic or diagnostic misconceptions in research [46]. Understanding the strategies used to protect participants’ data is important not only for REC members but also, for research participants who are required to provide voluntary, informed consent where the collection, sharing and secondary use of their data is concerned [34, 45, 46]. This ethical imperative signifies an understanding of the conditions and implications of research participation; however, the language used in consent forms is not always technically sound and accurate [47]. Interviewees voiced concerns over the use of inadequate consent in data-intensive research which may include ambiguous language that veil intentions of secondary use of data without seeking data subject (re)consent and/or REC approval. Since AI algorithms can be applied to existing datasets to yield new information, consent may need to be secured again to repurpose data if the original consent no longer applies [12, 48]. However, transparency in how participants’ data will be used or similar processes may not be completely achieved, especially when data is used to develop complex and unexplainable algorithms in the future [48]. In addition, all possible future uses of data cannot be disclosed and may be unknown at the time of data generation [12, 48]. This makes procuring true ‘informed consent’ in these contexts challenging and, in the interim, unrealistic as such explanations play a pivotal role in one’s ability to make informed decisions about sharing their data [48].

The African Open Science Platform serves as a digital revolution that can accelerate big data research in SSA and foster open science, yet the increased adoption of open data policies requiring the sharing of de-identified individual-level health research data makes data governance even more important [48–52]. Rather than making data openly available without restrictions, a couple of interviewees advocated for a DAC to review and manage data access requests given the committee’s expected technical expertise and knowledge of data governance to better address challenges. Some interviewees reported that RECs were the ideal stakeholders for reviewing DTA’s given their members’ diverse skill sets, while others sided with legal entities [10, 15]. These interviewees further expressed concern that RECs may not be adequately qualified to perform such a task and that managing data access requests may overburden RECs with added responsibility. Consequently, the thoroughness of protocol review may be negatively affected by heavy workloads as documented in the literature [49, 50]. Although no widely accepted framework exists for the organisation and function of DACs [10], considering its significance as a governance mechanism in overseeing controlled data access, it would be advantageous for SSA research-based institutions to encourage and authorise the establishment of these committees [10, 51].

Similarly, the lack of adequate data sharing regulatory frameworks across SSA was firmly cited as a major challenge among interviewees. Where non-existent or poor data sharing legislation was reported, a lack of institutional policies and ethics guidance often accompanied the legal vacuum. One interviewee, in particular, emphasised the importance of regulation to foster meaningful knowledge co-creation, whereby local African researchers are equally involved in research collaborations and fairly acknowledged in resulting outputs. This finding concurs with other studies suggesting that most LMICs lack robust regulatory and governance structures [17, 52–55]. Such insufficiencies in catering to the lawful and safe sharing or cross-border transferring of data caused some interviewees to experience first-hand consequences of neo-colonial science, also known as helicopter research, through exploitative research practices. This is reflected in the literature and stresses the detrimental effects of inadequate structures [17, 54–58], which not only threaten to erode trust in science but also, ultimately expose vulnerable research participants to potential harm.

The alleviation of any harm to research participants should be a steady objective within RECs when reviewing data-intensive research protocols, particularly when the transfer of human biological samples and data is involved. In doing so, ensuring that such transfers are accompanied by legal agreements such as MTAs and/or DTAs between the sender and recipient is among the many REC responsibilities [59]. Interviewees were more familiar with an MTA than a DTA, which may potentially indicate that data sharing within some SSA institutions or countries occurs under the radar or merely operates under a Memorandum of Understanding (MOU), export permits and/or benefit-sharing arrangements during research collaborations.

Furthermore, while all interviewees recognised the importance of a DTA, opinions varied on its ideal structure. On the one hand, a portion of interviewees proposed combining the DTA and the MTA into one master document to simplify the transferral of both data and biological materials. This cohort echoes the recommendations of Mahomed *et al* who offers the idea of developing one consolidated document for the transfer of samples and data [60]. On the other hand, another portion of interviewees advocated keeping DTAs separate from MTAs given their differences in paradigms. This stance coincides with the views of Swales *et al*. who propose the idea of non-exclusive and accredited DTA templates [61].

Interviewees were divided between supporting a national and an institutional DTA. Of those who expressed a liking for a national DTA, some justifications included that a nationalised agreement would mitigate challenges arising from the several inequities, inconsistencies and irregularities present within various DTA templates from varying institutional practices. One interviewee proposed a nationalised DTA to reduce national reliance on guidelines or frameworks developed by other countries. As a result, the development of DTAs by SSA countries could be a step in the right direction for regulating data exchange between researchers or institutions. Other interviewees sided with the idea to pursue institutional DTAs which could be tweaked to the suitability of the research collaboration.

Furthermore, interviewees listed various key elements to be considered when developing a well-furnished and context-specific DTA. Frequent suggestions included the type of data being transferred, the storage location and duration, the data destruction process, data access management and security and the purpose of the data transfer. These suggestions mirror some of the many provisions to be incorporated into a DTA template offered by Mahomed and Labuschaigne to better safeguard participants‘ personal information and better guide RECs [62].

Despite our interviewees’ contradictory opinions on the ideal structure of a DTA, holistically engaging stakeholders such as researchers, RECs, research institutions, funders, community members and governments across SSA is crucial in supporting and fostering a mutual understanding between data recipients and data donors. Given the diverse contextual dynamics across SSA, DTAs will be shaped by their respective country’s laws and regulations. Furthermore, opinions on which stakeholders should be ideally responsible for initiating the developmental process of institutional or national DTAs varied slightly as most interviewees acknowledged multiple diverse stakeholders. To facilitate the seamless transfer and sharing of data from SSA countries across the region and globe, sustainable and efficient structures to regulate and secure data sharing, while simultaneously safeguarding study participants are crucial.

Fortunately, the African Union (AU) Data Policy Framework, endorsed in 2022, offers guidance in developing African national data systems to improve the utility and value derivation of data by promoting accessibility, sharing of benefits and the secure flow of data across the continent while also protecting human rights [63]. Furthermore, the AU framework provides guidelines for cross-border data transfer, ensuring that data is protected during the process. This fosters a synthesised data ecosystem and harmonised digital data governance systems.

### Study limitations

The study is not without its limitations and should be acknowledged when interpreting the findings. Interviews were only conducted in English although some interviewees were from Lusophone and Francophone African countries. This may have influenced their depth of articulation, yet they were still able to proficiently convey their ideas in English. We recommend that future studies include a translator during interviews with non-native English speakers, funding and resource permitting. Future studies would also benefit from involving more than one participant per country for a broader representation and better transversality to other RECs within a particular country. Another limitation to our study is selection bias as not all RECs or all SSA countries were approached. This was a baseline study to get an overall picture of SSA REC members’ perspectives on data sharing. Despite these limitations, this study provided valuable insights into the various perspectives and experiences of REC members regarding data sharing, particularly because most studies conducted in SSA primarily focused on researchers’ views. Although the challenges faced in reviewing data-intensive-related protocols were discussed, it was unclear how RECs managed these challenges and it would be useful for future studies to further explore this phenomenon and build on these findings.

## Conclusion

A lack of national data governance regulations and the unfamiliarity thereof by REC members as well as suboptimal research ethics training are contributing factors to data-intensive research challenges in LMICs. Current guidelines are not appropriate for offering valuable guidance to RECs and researchers. Scientists, researchers and scholars need to determine how to accommodate differences in carefully designed spaces to support consensual decision-making and collaborative knowledge creation. Building upon the current understanding of research data ecosystems, further research should also examine how different domains govern, organise and negotiate the management of shared data resources and how fast-evolving multidisciplinary, interdisciplinary and transdisciplinary fields disrupt, negotiate and transform this process. To promote efficient data sharing within and across SSA, guidelines that are lucid and comprehensive, inclusive of ethical, legal and social aspects, and encompass principles of openness, storage, sharing and secondary use are highly recommended. The development of such guidelines would require consultation with relevant stakeholders who possess expertise and experience in data-intensive research and data sharing to establish a management system to streamline processes and efficiency. Together with the establishment of comprehensive guidelines, the accreditation of REC members in the review of data-intensive protocols is also critical in promoting efficient data sharing on the continent.
